# Overexpression of *TaLBD16-4D* alters plant architecture and heading date in transgenic wheat

**DOI:** 10.3389/fpls.2022.911993

**Published:** 2022-09-21

**Authors:** Huifang Wang, Xiaofan Han, Xiaofeng Fu, Xinling Sun, Hailong Chen, Xirui Wei, Shubin Cui, Yiguo Liu, Weiwei Guo, Ximei Li, Jiewen Xing, Yumei Zhang

**Affiliations:** ^1^ Shandong Provincial Key Laboratory of Dryland Farming Technology, Qingdao Agricultural University, Qingdao, China; ^2^ State Key Laboratory for Agrobiotechnology, Key Laboratory of Crop Heterosis Utilization (MOE), Beijing Key Laboratory of Crop Genetic Improvement, China Agricultural University, Beijing, China

**Keywords:** *TaLBD16-4D*, plant architecture, heading time, auxin, wheat

## Abstract

Lateral organ boundaries domain (LBD) proteins, a class of plant-specific transcription factors with a special domain of lateral organ boundaries (LOB), play essential roles in plant growth and development. However, there is little known about the functions of these genes in wheat to date. Our previous study demonstrated that *TaLBD16-4D* is conducive to increasing lateral root number in wheat. In the present work, we further examined important agronomical traits of the aerial part of transgenic wheat overexpressing *TaLBD16-4D.* Interestingly, it was revealed that overexpressing *TaLBD16-4D* could lead to early heading and multiple alterations of plant architecture, including decreased plant height, increased flag leaf size and stem diameter, reduced spike length and tillering number, improved spike density and grain width, and decreased grain length. Moreover, auxin-responsive experiments demonstrated that the expression of *TaLBD16-4D* in wild-type (WT) wheat plants showed a significant upregulation through 2,4-D treatment. *TaLBD16-4D*-overexpression lines displayed a hyposensitivity to 2,4-D treatment and reduced shoot gravitropic response. The expressions of a set of auxin-responsive genes were markedly different between WT and transgenic plants. In addition, overexpressing *TaLBD16-4D* affected the transcript levels of flowering-related genes (*TaGI*, *TaCO1*, *TaHd1*, *TaVRN1*, *TaVRN2*, and *TaFT1*). Notably, the expression of *TaGI*, *TaCO1*, *TaHd1*, *TaVRN1*, and *TaFT1* displayed significant upregulation under IAA treatment. Collectively, our observations indicated that overexpressing *TaLBD16-4D* could affect aerial architecture and heading time possibly though participating in the auxin pathway.

## Introduction

Common wheat (*Triticum aestivum* L.) is one of the major globe cereal crops supplying approximately 20% of the daily calories and proteins for the world’s population (Shiferaw et al., 2013; Tucker et al., 2017). Increasing grain yield of wheat is a major global challenge to provide sufficient food for the growing world population (Grassini et al., 2013; Jiang et al., 2017). To produce high yield of wheat, it needs not only the high yield potential determined by yield components (grain number per spike, number of fertile tillers per plant, and thousand grain weight) (Xue et al., 2008; Zhang et al., 2018) but also ideal plant architecture of morphological traits such as root system, plant height, branch or tiller number and angel, and flag leaf size and angle (Rogers and Benfey, 2015; Li et al., 2019). Of these, plant height is one of the most prominent architecture traits in crop plants, because of its role in planting density, harvest index, and lodging resistance that are closely associated with crop yield potential. Leaf size, especially flag leaf size, is a major contributor to grain yield in cereal crops, as it affects photosynthetic efficiency, carbohydrate synthesis, accumulation, and partitioning (Biswal and Kohli, 2013; Shahinnia et al., 2016). Also, plasticity of heading date (or flowering time) in wheat plays an important role in adaptation as it enables the regulation of plant development in different environments and guarantees the broad geographical range of wheat varieties. Selection for the optimum heading date to adapt their local growing conditions has also contributed to the increase of wheat yields globally (Kamran et al., 2014; Chen et al., 2020).

The molecular mechanisms regulating yield, plant architecture, and heading date have been extensively investigated and were demonstrated to be regulated by sophisticated phytohormone signaling pathways (Wang and Li, 2006; Ivanchenko et al., 2008; Hao et al., 2013; Renau-Morata et al., 2021). In particular, auxin plays critical roles in many, if not all, plant growth and developmental processes including apical dominance, phototropic and gravitropic responses, stem and leaf growth, flower development, and lateral and adventitious root formation (Okushima et al., 2005a; Vanneste and Friml, 2009; Kim et al., 2020). The auxin signaling pathway is tightly controlled at the cellular and tissue level mainly by transcription factors (Feng et al., 2012; Zhang et al., 2020). The LBD transcription factor is plant-specific and defined by the LOB domain, which has been designated to this functional group based on its nuclear localization and capacity to bind to DNA motifs (Husbands et al., 2007; Majer and Hochholdinger, 2011). The *LBD* family is composed of 42 members in *Arabidopsis*, 35 members in rice, 44 members in maize, and 24 members in barley while 94 members have been identified in wheat (Yang et al., 2006; Zhang et al., 2014; Guo et al., 2016; Xu et al., 2021). According to the structure of the LOB domain, the *LBD* family is classified into class I and class II subfamily. Class I protein members contain a zinc finger-like domain and a Leu-zipper-like coiled-coil motif, while class II members only have a conserved zinc finger-like domain (Landschulz et al., 1988; Shuai et al., 2002). The majority of *LBD* genes are expressed at the adaxial base of plant lateral organs and play an important role in the formation and development of plant lateral organs as well as metabolism in plants (Shuai et al., 2002; Chalfun-Junior et al., 2005).

Up to date, some members of the *LBD* family have been functionally identified in different species. Class I *LBD* genes included *AtLBD16*, *AtLBD18*, and *AtLBD29* that not only participate in auxin-dependent lateral and adventitious root formation downstream of *AtARF7* and *AtARF19* (Okushima et al., 2005b; Lee et al., 2009; De Smet et al., 2010) but also act as key regulators of callus formation in plant regeneration (Okushima et al., 2007; Fan et al., 2012). Transcript levels of *AtDDA1*/*AtLBD25* are reduced by exogenous indole-3-acetic acid (IAA or auxin) treatment, and the *dda1* mutant exhibits fewer lateral roots and aberrant hypocotyl elongation in the dark (Mangeon et al., 2011). *ASYMMETRIC LEAVES2* (*AS2*)/*LBD6* functions in the establishment of leaf polarity by repression of cell proliferation in the adaxial domain of *Arabidopsis* leaves (Semiarti et al., 2001; Iwakawa et al., 2007). *AtLBD30* is required for auxin-mediated development of embryogenesis and lateral organs (Rast and Simon, 2012). In maize, *Ramosa2* (*Ra2*, an ortholog of *AtLOB*) is involved in floral development (Bortiri et al., 2006). The *indeterminate gametophyte1* (*ig1*) gene of maize, encoding a LOB domain protein with high similarity to *ASYMMETRIC LEAVES2* of *Arabidopsis*, affects leaf development as well as embryo sac development (Evans, 2007). In rice, *OsCRL1* (an ortholog of *AtLBD29*) regulates crown and lateral root formation relying on the auxin signaling pathway (Inukai et al., 2005). *OsLBD3-7* acts as an upstream regulator of bulliform cell development to regulate leaf rolling (Li et al., 2016). *OsIG1* encodes a LOB domain protein and regulates floral organ and female gametophyte development (Zhang et al., 2015). Among class II *LBD* genes, *AtLBD37*, *AtLBD38*, and *AtLBD39* respond to exogenous nitrate and function in anthocyanin synthesis and nitrate metabolism (Rubin et al., 2009). *AtLBD41* participates in leaf dorsoventral determination (Chalfun-Junior et al., 2005). *OsLBD37* and *OsLBD38* delay the heading date by repressing the expression of the key regulator Ehd1 as well as the florigen genes *Hd3a* and *RFT1* and improve plant height and grain numbers and yield in overexpression plants (Li et al., 2017). Although extensive investigations of the *LBDs* in different species have provided better understanding of this gene family, the functional conservation and diversity of *LBD* genes in wheat remain largely unknown.

In our previous work, we found that *TaLBD16* (an ortholog of *AtLBD16*) contributes to the wide variation of lateral root number during wheat evolution, and overexpression of *TaLBD16-4D* can significantly increase lateral root number in wheat (Wang et al., 2018). As a continuation of this work, the present study focused on the function and molecular mechanism of *TaLBD16-4D* in the aerial part of wheat. Our results demonstrated that constitutive overexpression of *TaLBD16-4D* led to pleiotropic effects on plant architecture, as well as early heading. Moreover, *TaLBD16-4D* overexpression plants displayed less sensitivity to 2,4-D treatment and decreased gravity response. Our findings showed that these changes could be ascribed to the attenuated auxin pathway in *TaLBD16-4D* overexpression plants. The identification of *TaLBD16-4D* can not only extend our understanding of the molecular and genetic regulation of wheat heading date and plant architecture but also serve as a new potential genetic regulator for breeding high yield wheat cultivars.

## Materials and methods

### Plant materials and growth conditions

Our previous research of *TaLBD16-4D* (Wang et al., 2018) provided *TaLBD16-4D*-overexpression transgenic wheat lines (OE1 and OE2) and wild-type (WT) Fielder for this study. For experiments in the greenhouse, the sterilized seeds of transgenic wheat plants and WT were incubated at 4°C for 3 days in the dark and then cultured at room temperature under dark conditions for 12 h. Uniformly germinated seeds were grown in a greenhouse with a 16-h/8-h light/dark photoperiod, a light intensity of 14,100 lx, a temperature regime of 24:18°C (light:dark), and 60% humidity. For field trials, transgenic wheat plants and WT used to evaluate yield-related traits were planted with three replicates under natural field conditions during long days in the region of Beijing (40.14°N, 116.19°E), and each replicate contained two rows that were 1.5 m long and 0.3 m apart with a sowing rate at 25 seeds per row. The irrigation and other management of field trials were in accordance with local standard practices.

### Evaluation of agronomic characteristics and yield traits

The WT wheat plants and transgenic lines were planted in the experimental field with three replicates. For each replicate, the heading date (HD) of each genotype was calculated as days from the sowing date to the date when approximately 50% spikes were visible. Agronomic traits, including plant height, flag leaf length and width, spike length, and spikelet density, were examined before harvest. After maturity, grain number per spike (GNS) was given by 15 main spikes. Effective tillers of each plant were counted to determine the total number of wheat ears in each plant from 15 individual plants. Grain yield per plant was estimated by 10 plants. Thousand-grain weight (TGW), grain length (GL), and grain width (GW) were determined using a camera-assisted phenotyping system (Wanshen, Hangzhou).

### Measurement of free IAA content

Uniformly germinated seeds from WT and transgenic wheat lines were grown in a greenhouse for 2 days. Next, the 2-day-old seedlings were transplanted to a culture box filled with 1/5th strength Hoagland solution. Solution was changed every 2 days. The endogenous content of IAA was quantified according to the method described by Yue et al. (2019). The roots and aerial part of WT and OE lines were collected respectively at 12 days after germination, frozen in liquid nitrogen, and stored at -80°C. For the extraction of free IAA, a 0.1-g sample was soaked with 4 ml extracting solution and shaken in the dark for 12 h at 4°C. Then, the sample was centrifuged at 10,000 rpm for 15 min at 4°C. The supernatant was removed to a new 10-mL tube with 3 mL extracting solution and stored at 4°C in darkness. The remaining sediment was reextracted twice for 30 min in the dark at 4°C. After reextraction every time, the sample was centrifuged again at 10,000 rpm for 15 min at 4°C. The three resulting supernatants were merged together (8 ml), placed on ice, and dried with nitrogen in the dark. Next, the dry sample was redissolved with 0.8 ml methanol. The powder of IAA (Sigma-Aldrich) was dissolved with different concentrations of methanol to construct the calibration curve. Finally, the above supernatants and standard solution were filtered with a 0.22-nm filter membrane. The determination of IAA was performed by Agilent Technologies 6400 high-performance liquid chromatography (HPLC). One milliliter of sample was used for detection. Each sample was analyzed with three biological replicates.

### Auxin treatment at the seedling stage

The 2-day-old seedlings of WT and OE lines with the same vigor were transferred to a culture box filled with 1/5th strength Hoagland solution for 5 days in the greenhouse. The culture solution was changed every 2 days. Then, the 7-day-old uniform seedlings were treated with different concentrations of 2,4-D (0.001, 0.01, 0.1, 1, and 10 μM) and 1/5th strength Hoagland solution for another 5 days. Maximum root length was measured from the root tip to the root–shoot junction. Shoot length was measured from the tip of the longest leaf to the root–shoot junction. Shoot fresh mass was assessed using an automated electronic scale. The experiment was repeated three times, with at least eight plants per treatment group. For auxin-related gene expression analysis, leaves and root tissue were collected from the 5-day-old seedlings after 30 μM 2,4-D treatment for 0 or 12 h. For flower-related gene expression analysis, the 7-day-old uniform seedlings were cultured with 1/5th strength Hoagland solution, until the seedlings at the three-leaf stage were treated with 0.1 μM IAA for 0, 12, 16, or 24 h. The leaves at the three-leaf stage were used for gene expression analysis.

### RNA extraction and reverse transcription qPCR analysis

Total RNA was extracted using the standard TRIzol RNA isolation protocol (Vazyme Biotech, Nanjing, China), according to the manufacturer’s instructions. The RNA samples were digested with purified DNase I, and first-strand cDNA synthesis was performed using HiScript II One-Step RT-PCR Kit (Vazyme Biotech, Nanjing, China). Reverse transcription qPCR (RT-qPCR) was conducted using SYBR Color qPCR Master Mix (Vazyme Biotech, Nanjing, China) with the QuantStudio 3 Real-Time Fluorescence Quantitative PCR System (Thermo Fisher scientific, Waltham, USA). The RT-qPCR conditions and analytical methods were the same as those described by Wang et al. (2018). The specific gene primers for qPCR were designed according to the conserved region of three homeologs. The wheat *Actin* gene was used as an internal control (Shimada et al., 2009). The accession numbers of *TaActin* homoeologous genes were TraesCS1A02G274400, TraesCS1B02G283900, and TraesCS1D02G274400. Each sample was quantified in triplicate. A description of the genes and primer sequences is given in **Supplementary Table 1**.

### Shoot gravitropic response analysis

Uniformly germinated seeds from WT and *TaLBD16-4D* OE lines were placed vertically on 0.6% agar medium for 2 days in the greenhouse. The 2-day-old seedlings were reoriented by 90° and grown for another 2 days. The shoot curvature was measured as the shoot gravitropic response. Each genotype was performed with three biological replicates, and the number of seedlings for each replicate was at least 10.

### Statistical analysis

A one-way analysis of variance (ANOVA) with the least significant difference (LSD) test was conducted using IBM SPSS 19.0 for Windows (IBM, Armonk, NY, USA). Student’s *t*-test was conducted using EXCEL 2019. All statistical tests were performed by two-sided significance tests with a 0.05, 0.01, or 0.001 significance level.

## Results

### Ectopic expression of *TaLBD16-4D* causes multiple phenotypic variations in wheat

**Figure 1:**
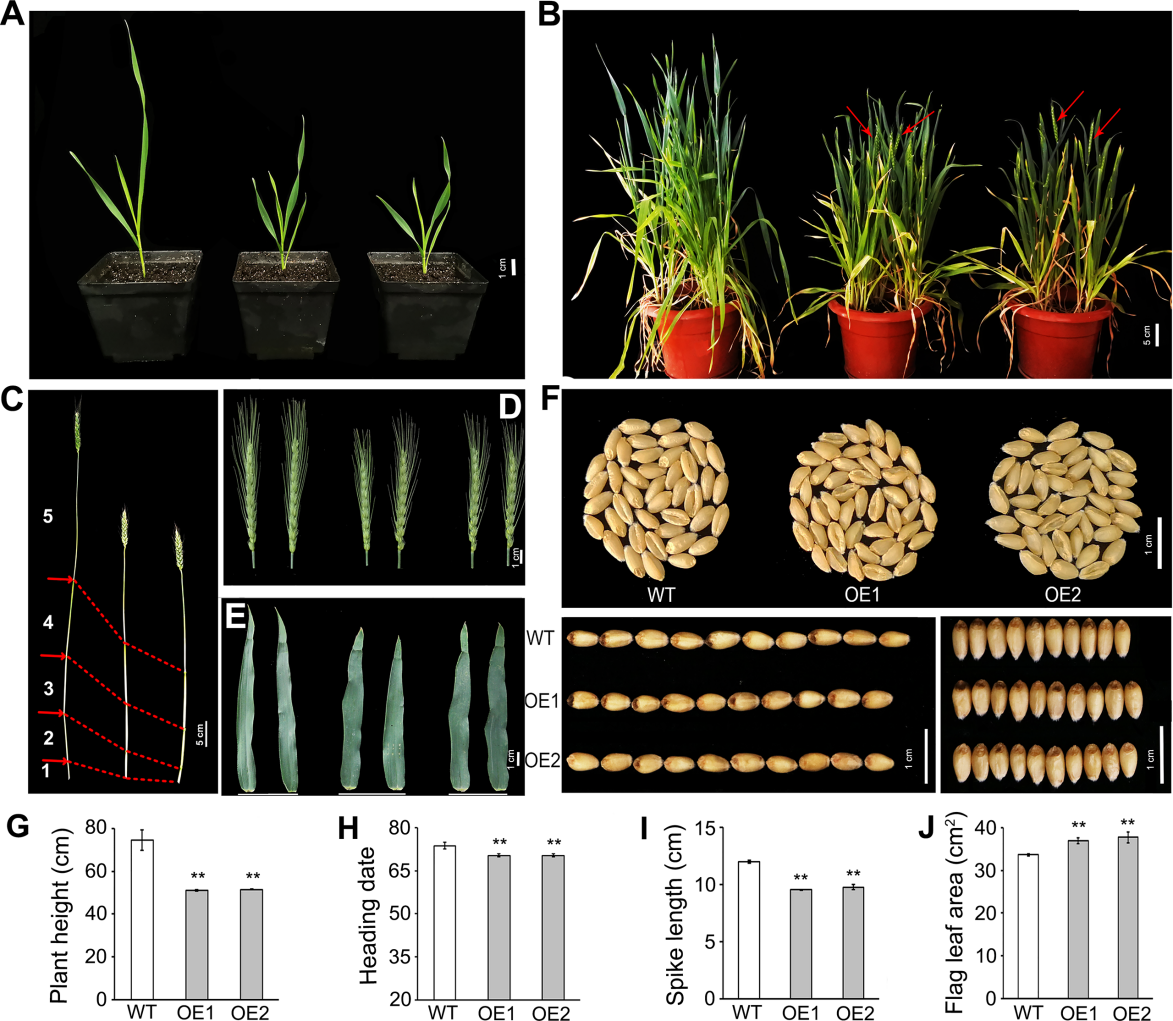
Pleiotropic effects of overexpressed *TaLBD16-4D* on wheat development. Comparisons of several important traits between the two *TaLBD16-4D*-overexpression transgenic plants (OE1 and OE2) and wild type (WT). **(A)** Photos of WT (left) and two OE lines (middle and right) taken when OE lines reached the tillering stage. **(B)** Phenotypic characteristic of WT (left) and two OE lines (middle and right) taken when OE lines reached the heading stage. Red arrow heads indicate spikes at the heading stage. **(C)** Main culms of WT (left) and two OE lines (middle and right). **(D)** Main spikes of WT (left) and two OE lines (middle and right). **(E)** Flag leaf of WT (left) and two OE lines (middle and right). **(F)** Morphology of grains in the WT and two OE lines. Comparison of plant height **(G)**, heading date **(H)**, spike length **(I)**, and flag leaf area **(J)** in WT and two OE lines, respectively. All values in G–J are the mean ± SD (n = 3). Statistical differences between WT and two OE lines are indicated by asterisks and were determined by Student’s t-test and **P < 0.01.

Overexpression lines of *TaLBD16-4D* were previously generated with ORF driven by the maize *ubiquitin* (*Ubi*) promoter (Wang et al., 2018). In the present study, two homozygous overexpression (OE) lines (OE1 and OE2) in T_4_ were used to elucidate the function of *TaLBD16* in the aerial part. At the seedling stage, various traits of OE1 and OE2 differed significantly from those of the wild type (WT) under greenhouse conditions, including shoot length, leaf width, leaf length, leaf area, total chlorophyll content (SPAD), shoot fresh weight, and growth rate. The shoot height and leaf length in lines OE1 and OE2 were both significantly lower than those in the wild type (**Figure 1A**; **Supplementary Figures 1A-C**), whereas the leaf width and total chlorophyll content (SPAD) in lines OE1 and OE2 were significantly higher (**Supplementary Figures 1A, D, F**). Furthermore, the leaf area and shoot fresh weight in OE lines were slightly lower than WT (**Supplementary Figures 1A, E, G**). In addition, overexpression of *TaLBD16-4D* accelerated the growth rate at the seedling stage, that is, OE1 and OE2 had entered the tillering stage when the wild type grew to the three-leaf stage (**Figure 1A**).

To further investigate the effects of *TaLBD16*-*4D* on yield and yield-related traits, we compared the traits between *TaLBD16-4D*-overexpression lines and WT under field conditions after the seedling stage. The most noticeable phenotype is the reduction of plant height, consistent with a decrease at the seedling stage. The plant heights of two OE lines were reduced by 31.38% and 31.02%, respectively (**Figures 1B,G**). Since wheat plant height is primarily determined by stem internode length, we measured the stems and internodes of transgenic lines. The results showed that the internode number in transgenic lines was one less than that in WT; at the same time, OE lines displayed significantly shortened internodes (**Figure 1C**; **Supplementary Figure 2**). These results revealed that overexpressing *TaLBD16-4D* led to decreased plant height of wheat by shortening the internode length together with the low node number. In addition, transgenic lines headed significantly early by 3.34 days, and showed reduced spike length by 20.45% and 15.83%, and increased spike density by 11.46% and 12.05%, respectively (**Figures 1B, D, H, I**; **Table 1**). Interestingly, the flag leaf width in OE lines was markedly higher, but flag leaf length in OE lines was much lower than WT, consistent with the phenotype of the first leaf at the seedling stage (**Figure 1E**; **Table 1**; **Supplementary Figures 1C, D**). Totally, the flag leaf area increased by 9.78% and 12.13% in the two OE lines, respectively (**Figures 1E, J**). Compared with the WT, the thousand-grain weights (TGW) of two OE lines were similar with those of WT, which might result from a trade-off between grain width increased by 3.7%~5.7% and grain length decreased both by 3.6%. Further examination showed that the grain yield per plant of both two overexpression lines were reduced by 23.11% and 33.56%, respectively, compared with the WT (**Table 1**), primarily because of lower grain number per spike and productive tillers per plant (**Table 1**).

**Table 1:** Measurements of yield and related-yield traits in the *TaLBD16-4D* transgenic and wild-type plants.

Traits	WT	OE1	OE2
Number of spikelets per spike	19.89 ± 0.29 (A)	17.60 ± 0.26 (B)	18.10 ± 0.44 (B)
Spike density	1.66 ± 0.04 (A)	1.85 ± 0.03 (B)	1.86 ± 0.08 (B)
Flag leaf width	1.84 ± 0.05 (B)	2.19 ± 0.03 (A)	2.26 ± 0.05 (A)
Flag leaf length	18.33 ± 0.55 (A)	16.83 ± 0.39 (B)	16.72 ± 0.53 (B)
Number of productive tillers per plant	14.03 ± 2.82 (A)	10.16 ± 0.24 (B)	9.63 ± 1.43 (B)
Grain number per spike on main stem	57.10 ± 1.27 (A)	45.77 ± 1.02 (B)	43.63 ± 1.86 (B)
Grain length	6.19 ± 0.12 (A)	5.97 ± 0.04 (B)	5.97 ± 0.02 (B)
Grain width	2.96 ± 0.01 (B)	3.07 ± 0.01 (A)	3.13 ± 0.01 (A)
Thousand grain weight	34.32 ± 0.12 (A)	33.51 ± 0.21 (A)	34.05 ± 0.45 (A)
Grain yield per plant	16.09 ± 1.40 (A)	12.37 ± 0.77 (B)	10.69 ± 2.58 (B)

A, B, ranked by LSD test at P ≤ 0.01.

### *TaLBD16-4D* affects stem and leaf growth and development

**Figure 2:**
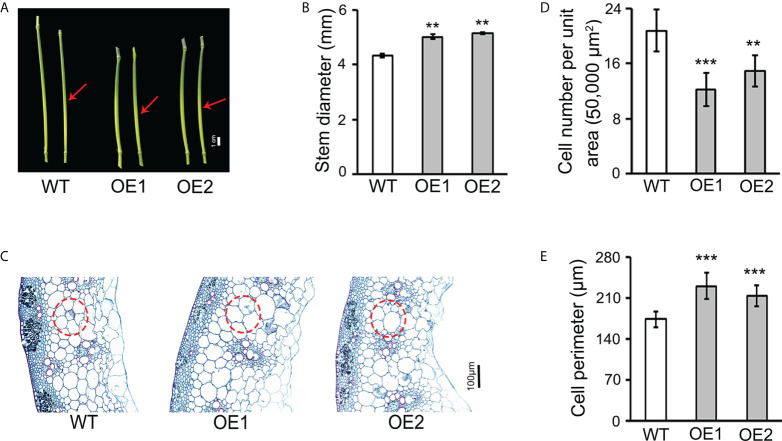
Overexpression of *TaLBD16-4D* altered wheat stem development. **(A)** Phenotypes of the fourth nodes and stems above the ground from the main culms for WT and *TaLBD16-4D*-overexpression plants grown in the field under natural long-day conditions. Arrowheads indicate the middle region of the fourth stem node used for observation. **(B)** Stem diameter of two OE lines and WT plants. **(C)** Transverse section analysis of the fourth stem node of *TaLBD16-4D-*overexpression and WT plants. Red circles indicate the major cell types selected for cell counting and measurement. **(D)** Cell number per unit area in c. **(E)** Cell size in c. For cell number (per unit area) counting, a total area of 50,000 µm^2^ for each sample was investigated. For cell perimeter determination, representative cells within each of the red circle regions were selected for cell size measuring. Data reported are shown as mean ± SD (n = 7). Asterisks indicate statistically significant differences between WT and *TaLBD16-4D*-overexpression plants determined using Student’s *t*-test. ** and *** indicate significant differences at the 0.01 and 0.001 levels, respectively.

*TaLBD16-4D* OE lines also exhibited multiple phenotypes related to stem and leaf. Compared with the WT, the two OE lines had fewer nodes and shortened internodes (**Figure 1C**; **Supplementary Figure 2**) but increased the thickness of the stem by 15.17% and 18.16%, respectively (**Figures 2A, B**). To investigate the effect of *TaLBD16-4D* at a cellular level, we performed paraffin sectioning of stem and leaves of these plants. Transverse histological sectioning analysis of the fourth stem internode showed significantly reduced cell numbers per unit area in OE1 (-41.47%) and OE2 (-28.41%) when compared with WT (**Figures 2C, D**), suggesting that cell sizes of the two OE lines were altered. Direct cell measurement exhibited that the cell perimeters were significantly increased in OE1 (+32.27%) and OE2 (+22.57%) (**Figures 2C, E**). In addition, leaf widths of both OE lines were significantly increased but leaf lengths were markedly reduced (**Figure 1E**; **Supplementary Figure 1**). Consistent with the transverse sectioning results of stems, transverse sectioning of the first leaf confirmed the significantly increased cell size (+10.22% in OE) and reduced cell numbers per unit area (-16.82% in OE) (**Supplementary Figures 3A, C, D**). Longitudinal sectioning of these leaves showed significantly reduced cell length in OE (-9.44%; **Supplementary Figures 3B, E**), which might cause decreased leaf length in transgenic lines. Notably, the OE plants had advanced vascular systems and parenchyma (**Figure 2C**; **Supplementary Figure 3A**), indicating an increased transport capacity and stores of photosynthate, which are crucial for grain weight. These results suggested that overexpression of *TaLBD16-4D* enhanced the size of stem diameter and leaf width probably by promoting cell enlargement. Indeed, although the OE plants were much shorter, the thousand grain weight of the two OE lines was almost identical to that of the WT.

### *TaLBD16-4D* is involved in the auxin pathway and increased the IAA concentration

**Figure 3:**
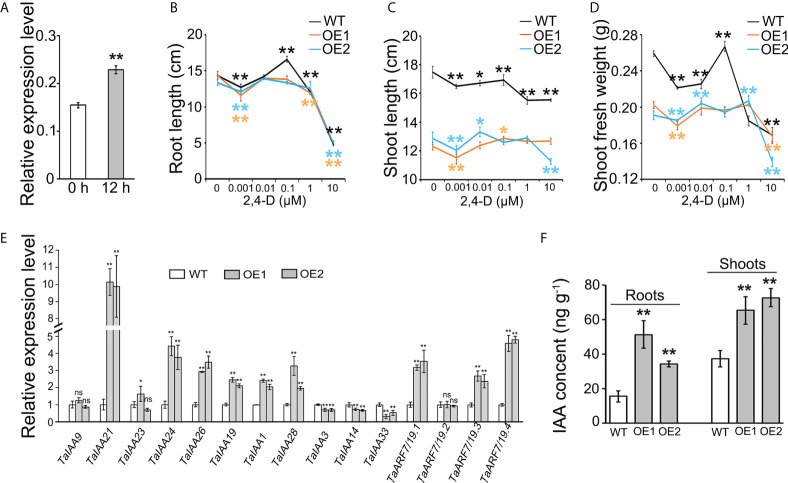
Effects of *TaLBD16-4D*-overexpressed wheat plants on the auxin pathway. **(A)** Expression patterns of the *TaLBD16-4D* gene in roots of WT under 2,4-D treatment. Five-day-old seedlings grown in a hydroponic culture were treated with 30 μM 2,4-D for 0 or 12 h. The expression of *TaActin* was used to normalize mRNA levels. The values are means ± SD of three biological replicates. Comparison of root length **(B)**, shoot length **(C)**, and shoot fresh mass **(D)** between *TaLBD16-4D* overexpression lines (OE1 and OE2) and WT with different concentrations of 2,4-D at the seedling stage. The 2,4-D concentrations were 0, 0.001, 0.01, 0.1, 1, and 10 μM, respectively. Three independent biological replicates were used (eight plants per biological replicate). Values are indicated as mean ± SD. **(E)** Expression analysis of 16 auxin signaling-related genes in the leaves of WT, OE1, and OE2, determined using qRT-PCR. The relative levels of each gene were normalized with the respective gene set as 1. *TaActin* was used as the internal standard. **(F)** Quantification of the IAA levels in the aerial part of WT and OE lines at the seedling stage. All values in d and e are indicated as mean ± SD (n = 3). Statistical differences between WT and two OE lines are indicated by asterisks and were determined using Student’s *t*-test ns, not significant; *P < 0.05; **P < 0.01.

In *Arabidopsis thaliana*, *AtLBD16* is directly activated by auxin *via* AUXIN RESPONSE FACTOR 7 (ARF7) and ARF19 (ARF7/19) to induce the initiation of lateral root primordium (Okushima et al., 2007). Our previous study documented that the altered expression of *TaLBD16-4D* promotes the formation of lateral root (Wang et al., 2018). Synthetic auxins, such as 1-NAA, 2,4-D, and picloram, have been most commonly used for the identification of auxin receptors, auxin transport carriers, transcription factor response to auxin, and cross talk among phytohormones, which are not subject to the many endogenous homeostatic and metabolic mechanisms that can affect IAA (Ljung et al., 2002). Synthetic auxins, including 2,4-D, can induce strong changes in expression of auxin-related genes that ultimately lead to auxin-insensitive response in the cereal seedling roots and/or shoots (Bian et al., 2012; Xu et al., 2017; Guo et al., 2021). Thus, it was speculated that the *TaLBD16-4D* gene might be involved in auxin-mediated growth events in wheat. To test this hypothesis, we analyzed the *TaLBD16-4D* gene expression patterns of WT after exogenous 2,4-D treatment *via* qRT-PCR. As shown in **Figure 3A**, the expression of the *TaLBD16* gene in root of the WT was significantly upregulated by 2,4-D treatment compared to that of the control.

To further elucidate the relationship between *TaLBD16-4D* gene and auxin, we treated two OE lines and WT with different concentrations of synthetic auxin (2,4-D). Seven-day-old seedlings were transferred to a nutrient solution containing various concentrations of 2,4-D for another 5 days. The maximum root lengths of the overexpression plants and WT were significantly inhibited by 0.001 and 10 μM of 2,4-D. However, no obvious changes in OE lines were displayed under 0.01 and 0.1 μM 2,4-D treatment, compared to untreated OE lines (**Figure 3B**). By contrast, the WT plants showed more sensitivity of accelerating effect to 2,4-D application than the OE lines, especially with a major change in maximum root length in response to 0.1 μM 2,4-D (**Figure 3B**). We also examined the shoot length and shoot fresh weight of WT and OE lines in response to 2,4-D. The shoot length of WT significantly decreased with increasing 2,4-D concentration compared to the untreated control (**Figure 3C**), while the two OE lines showed less sensitivity to 2,4-D application than that of WT, with only a minor change in root length in response to increasing 2,4-D concentrations from 0.01 to 1 μM (**Figure 3C**). Moreover, the shoot fresh mass of two OE lines also exhibited a minor change compared to that of WT which showed a drastic decrease trend under 0.001, 0.01, and 1 μM 2,4-D (P < 0.01; **Figure 3D**). These data indicated that *TaLBD16-4D*-overexpression plants were less sensitive to auxin than WT.

The auxin signaling pathway in *Arabidopsis* consists of ARFs and the AUXIN/INDOLE-3-ACETIC ACID (Aux/IAA) repressor family. At a low auxin level, ARF7/19 form a protein complex with AUXIN/INDOLE-3-ACETIC ACID (Aux/IAA) proteins, which represses the transcriptional activation activity of ARF7/19. In contrast, high accumulation of auxin induces the proteolysis of Aux/IAAs to activate ARF7/19-dependent transcription (Ito et al., 2016). To further identify the role of *TaLBD16-4D* in the auxin pathway in wheat, we investigated the expression patterns of auxin-responsive genes orthologous to *Arabidopsis* genes involved in the auxin signal transduction pathway, including 11 *TaAux/IAAs* (Qiao et al., 2017) and four *TaARF7/19s* (Wang et al., 2018). Of these genes, six *TaAux/IAAs* and three *TaARF7/19s* were significantly upregulated in the young leaves of *TaLBD16-4D* overexpression plants compared to WT (**Figure 3E**). Notably, the relative mRNA abundances of *TaIAA3*, *TaIAA14*, and *TaIAA33* in two OE lines were less than those in WT (**Figure 3E**). In addition, in comparison with WT, the IAA contents were significantly increased in both roots and aerial part of *TaLBD16-4D* overexpression plants (**Figure 3F**). Interestingly, the expression levels of genes involved in auxin biosynthesis (*TaTAR2.1*, *TaYUCCA1*, *TaYUCCA7*, *TaYUCCA8*, *TaSUR36*) and transport (*TaPIN*) were significantly upregulated in both leaves and roots of two OE lines (**Supplementary Figure 4**). Together, these data suggested that *TaLBD16-4D* might function as an important regulator impacting on auxin signaling, biosynthesis, and transport.

Previous studies revealed that the presence of auxin response elements (AuxREs) in the promoter region of *TaLBD16* orthologous genes in rice, *Arabidopsis*, and maize are crucial for their function in root development (Inukai et al., 2005; Okushima et al., 2007; Taramino et al., 2007). Thus, we decided to analyze the putative AuxREs in the promoter region of three *TaLBD16* homoeologous genes (*TaLBD16-4A*, *TaLBD16-4B*, and *TaLBD16-4D*). Based on the Chinese Spring reference genome, three identical AuxREs were detected among the promoters of these three homoeologous genes. In addition, one extra AuxRE was detected in the promoter of *TaLBD16-4B* (**Supplementary Figure 5**).

### Overexpression of *TaLBD16-4D* impairs shoot gravitropism in wheat

**Figure 4:**
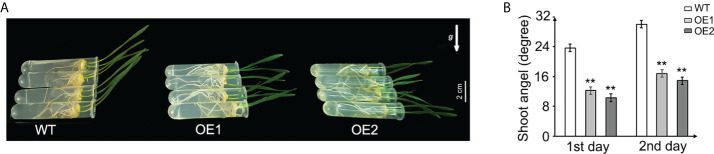
Gravitropism analysis of the *TaLBD16-4D*-overexpressing and WT plants. **(A)** Three-day-old seedlings of *TaLBD16-4D*-overexpressing (OE1 and OE2) and WT plants were grown vertically for 2 days and then horizontally grown for another 2 days. The arrow represents the direction of gravity. **(B)** The shoot angle of WT and *TaLBD16-4D*-overexpressed plants on the first day and second day. Three independent biological replicates were used (at least 10 plants per biological replicate). Values are mean ± SD (n = 3). Statistical differences between WT and two OE lines are indicated by asterisks and were determined using Student’s *t*-test: **P < 0.01.

Gravitropic response in shoot is also a typical auxin-related phenotype, which is associated with polar auxin transport leading to asymmetrical auxin distribution and differential growth between the upper and lower sides of responding organs (Li et al., 2007; Strohm et al., 2012; Song and Xu, 2013). To assess the gravity response of *TaLBD16-4D*-OE seedlings, the shoot angles of transgenic plants and WT were measured and compared. The seedlings grew vertically for 2 days after germination, and then the 2-day-old seedlings were placed horizontally for another 2 days. We analyzed the gravitropic curvature in these shoots on the first day and second day. The results demonstrated that *TaLBD16-4D* transgenic plants showed impaired gravitropic responses compared with WT (**Figure 4A**) and the gravitropic curvature of the two OE lines in each day was both significantly smaller than those of the wild-type seedlings (**Figure 4B**), indicating that overexpression of *TaLBD16-4D* impaired gravitropic responses of the seedling shoot.

### Overexpressing *TaLBD16-4D* promotes wheat heading *via* influencing the expression of flowering-time genes

**Figure 5:**
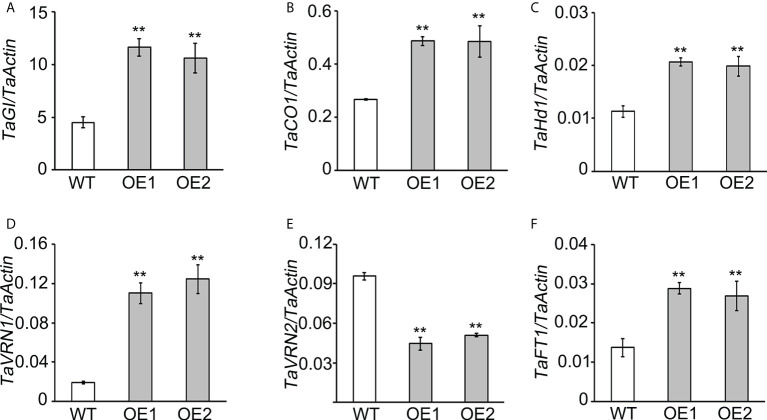
The expression analysis of flowering-related genes in WT plants and *TaLBD16*-*4D*-overexpressing transgenic lines. Expression levels of wheat flowering-related genes, including *TaGI* **(A)**, *TaCO1* **(B)**, *TaHd1* **(C)**, *TaVRN1* **(D)**, *TaVRN2* **(E)**, and *TaFT1* **(F)**, were determined by quantitative reverse transcription PCR (qRT-PCR). The third fully unfolded leaf at the three-leaf stage grown under long-day conditions. *TaActin* was used as an internal control for calculating the relative expression levels of the above genes. Values are shown as mean ± SD (n = 3). Statistical differences between WT and two OE lines are indicated by asterisks and were determined by Student's *t*-test **P < 0.01.

Changes in gene expression are often associated with phenotypic variation (Fay and Wittkopp, 2008). Given that *TaLBD16-4D* transgenic plants displayed early heading under long-day conditions (**Figures 1B, H**), we assumed that the expression of flowering-time genes might be influenced by *TaLBD16-4D*. In wheat, the heading is associated with the timing of floral transition, an important character in adapting to complicated environmental conditions (Cockram et al., 2007; Hu et al., 2020). The current consensus is that flowering promotion is subject to the vernalization and photoperiod pathways in wheat (Shim and Imaizumi, 2015). To investigate the roles of *TaLBD16-4D* in floral transition, we compared the expression patterns of six flowering-related genes (*TaGI*, *TaCO1*, *TaHd1*, *TaVRN1*, *TaVRN2*, and *TaFT1*) in leaves of the *TaLBD16-4D*-overexpression lines and WT at the three-leaf stage under long-day conditions. The analysis revealed that the two OE lines exhibited significantly upregulated expressions of *TaGI*, *TaCO1*, *TaHd1*, *TaVRN1*, and *TaFT1* and a downregulated expression of *TaVRN2* compared to WT (**Figure 5**). These results were consistent with the genetic network of wheat flowering-time genes in previous studies (Shimada et al., 2009; Hu et al., 2020) and also indicated that LBD16 might be important for floral transition acting upstream of the network.

To examine whether the expression profiles of flowering-related genes would be induced by auxin, qRT-PCR molecular assays were performed with the leaves of WT treated with IAA. We found that *TaGI*, *TaCO1*, *TaHd1*, *TaVRN1*, *TaVRN2*, and *TaFT1* exhibited significant dynamic expression changes in response to IAA treatment (**Supplementary Figure 6**). Meanwhile, the peak expression levels of these genes were different in response to IAA. Meanwhile, the peak expression levels of flowering-related genes were different in response to IAA. In brief, *TaGI* expression peaked at 16 h in response to IAA, while the transcripts of *TaCO1*, *TaHd1*, and *TaVRN2* peaked at 16 h. The expression of *TaVRN2* and *TaFT1* both peaked at 12 and 16 h in response to IAA (**Supplementary Figure 6**). These results suggested that flowering-related genes (*TaGI*, *TaCO1*, *TaHd1*, *TaVRN1*, *TaVRN2*, and *TaFT1*) in floral development processes might be regulated by auxin.

## Discussion

### Contribution of auxin to morphological changes by regulating the expression of the *TaLBD16-4D* gene in wheat

The phytohormone auxin is essential for plant growth and regulates a wide range of developmental processes. The underlying molecular mechanism of auxin signaling has been illuminated primarily in *Arabidopsis* (Song et al., 2009). A typical model for the auxin signal transduction pathway is that the Aux/IAA family interacts with auxin response factors (ARFs) at low auxin concentrations and inhibits the binding of ARF transcription factors to auxin-responsive elements (AuxREs) located in promoters of auxin-responsive genes, consequently repressing the expression of auxin-responsive genes (Ulmasov et al., 1997; Tian and Reed, 1999). At high auxin concentrations, auxin can promote the proteolytic degradation of the inhibitory Aux/IAA proteins by 26S proteasome (Tian et al., 2002; Kepinski and Leyser, 2004), and then ARFs are released from inhibition and regulate the transcription of auxin-responsive genes (Weijers and Jürgens, 2004). Previous studies have reported that several *LBD* genes, including *LBD16*, *LBD18*, *LBD29*, and *LBD33*, play crucial roles in auxin-regulated lateral root development in *Arabidopsis*. For instance, under high auxin levels, auxin derepresses the activity of the transcriptional activators ARF7 and ARF19 through the degradation of Aux/IAAs such as IAA3, IAA14, IAA15, IAA19, and IAA28 and consequently directly activates the transcription of downstream genes including *LBD16* and *LBD29* to regulate lateral root development (Okushima et al., 2005b; Kim et al., 2020).

Up to date, the mechanism underlying the relationship between *LBD* genes and auxin to control plant growth and development of the aerial part is poorly understood. Our previous study has demonstrated that *TaLBD16* is specifically expressed in roots and contributes to increase the lateral root number. Moreover, the D genome plays a crucial role in the increased lateral root number of allohexaploid wheats compared to their allotetraploid progenitors (Wang et al., 2018). Interestingly, there were some variations of the amino acids among the three *TaLBD16* homoeologous genes and the expression of *TaLBD16-4D* was much higher than those of 4A and 4B genomes in the seedling roots of natural and synthetic allohexaploid wheats, which might be responsible for functional divergence of three *TaLBD16* homoeologous genes (Wang et al., 2018). In this study, one more AuxRE was detected on the *TaLBD16-4B* promoter (**Supplementary Figure 5**), which indicated a higher sensitivity to auxin. Despite of this, the higher expression of *TaLBD16-4D* revealed that more external or internal factors along with auxin finally determined the divergences in the expression of *TaLBD16* homologues. Since the LBD family is composed of 94 members in wheat (Xu et al., 2021) and *TaLBD16-4D* includes 14 homologous members sharing a high possibility of functional redundancy of this gene with structurally related transcription factors (Wang et al., 2018), we decided to study its function by obtaining the overexpressing transgenic lines instead of the *Talbd16* mutant. Furthermore, no sequence variation was found between the D genome of *Triticum aestivum* L. and its diploid ancestor (*Ae. tauschii*) (Wang et al., 2018). Although there might be unexpected effects due to its ectopic expression, we believe that the overexpression strategy can also largely demonstrate the direct or indirect function of *TaLBD16-D*. Here, the function of *TaLBD16-4D* in aerial architecture was analyzed by obtaining ectopic overexpressing transgenic wheat lines. It is well established that regulation of root growth is under the control of several hormones and the expression of various transcriptional regulators (Lavenus et al., 2016). Auxin is an important mediator of long-distance signaling for organ–organ communication, which can move over long distances from root to shoot that plays an essential role in most aspects of plant growth and development (Molnar et al., 2010; Li et al., 2021). Notably, it was found that *TaLBD16*-*4D* played an important role in auxin-mediated multiplate traits in wheat. First, the expression of *TaLBD16*-*4D* was increased by 2,4-D (**Figure 3A**) and the endogenous IAA content in both shoots and roots of transgenic seedling was significantly higher than that of WT (**Figure 3F**); Second, three putative AuxREs in the promoter region of *TaLBD16*-*4D* were identified from CS, which may help the upstream TaARFs regulate the expression of the *TaLBD16-4D* gene (**Supplementary Figure 5**). Third, *TaLBD16*-*4D*-overexpressing plants displayed auxin-related phenotypes, such as dwarfism, wider and shorter flag leaf, and abnormal panicle morphology, under normal conditions (**Figure 1**). Fourth, *TaLBD16-4D*-overexpression wheat showed less sensitivity in root growth, shoot length, and shoot fresh weight when treated with 2,4-D (**Figures 3B–D**) and impaired shoot gravitropic response (**Figure 4**). Fifth, expressions of genes regulating auxin biosynthesis, transport, and signaling were significantly changed between transgenic wheat plants and WT (**Figure 3E** **;** **Supplementary Figure 4**).

### Influence of the *TaLBD16-4D* gene on heading time by the auxin-mediated photoperiodic pathway under long-day conditions

Wheat is a long-day plant grown widely across the world, and the differences in heading time among cultivars have led to a wide range of adaptability (Jackson, 2009; Shimada et al., 2009). Wheat cultivars that head early exhibit higher yield potential than cultivars that head later with the background of global warming, because the early-heading cultivars of wheat generally possess a longer post-heading and grain filling period and generate fewer total leaves per tiller but retain more green leaves and keep fewer leaves to senescence during anthesis (Tewolde et al., 2006). Heading time is tightly associated with the timing of floral transition. Numerous genetic studies in wheat have elucidated the regulatory network of heading-time (or flowering-time) genes involved in the vernalization and photoperiod pathway, which play important roles in floral induction (Worland, 2001; Kim et al., 2009; Song et al., 2015). *FLOWERING LOCUS T1*/*VERNALIZATION3* (*FT1/VRN3*) is an integrator of the vernalization and photoperiod pathways and functions as a flowering activator (Yan et al., 2006; Corbesier et al., 2007).

In the vernalization pathway, *VERNALIZATION1* (*VRN1*), *VERNALIZATION2* (*VRN2*), and *FT1/VRN3* are the key genes in wheat flowering regulation. *TaVRN2* is the flowering repressor to inhibit wheat flowering in winter by blocking the expression of *FT1* before vernalization (Yan et al., 2006; Li et al., 2011). The expression of *TaVRN1* is induced by vernalization, and it directly binds the promoters of *TaVRN2* and *TaFT1* to repress the expression level of *TaVRN2* and increase *TaFT1* expression to accelerate flowering (Yan et al., 2006; Deng et al., 2015). Although *TaVRN1*, *TaVRN2*, and *TaFT1* are naturally mutated in spring wheat varieties, there is a similar vernalization pathway of flowering regulation between spring wheat and winter wheat, ultimately promoting flowering under long-day conditions with the absence of vernalization (Fu et al., 2005; Kippes et al., 2016; Finnegan et al., 2018). The role of GA in *Arabidopsis* flowering has been studied, and it has been shown to regulate flowering *via* the FT pathway (Hisamatsu and King, 2008; Wang et al., 2016). Recently, growth evidence has shown that auxin is also involved in floral stage transition and flowering opening. In *Arabidopsis*, increased distribution of auxin and upregulation of auxin transporter genes delayed flowering in the near-null magnetic field (Xu et al., 2018). Auxin is also involved in the flowering pathway in strawberry through its regulation of *auxin response factor4* (*ARF4*) expression, which can bind to the promoters of the floral meristem identity genes *APETALA1* (*AP1*) and *FRUITFULL* (*FUL*), inducing their expression (Dong et al., 2021). The involvement of *TaCYP78A5* in auxin synthesis pathway and auxin accumulation contributes to the delayed heading and flowering in wheat (Guo et al., 2022). However, it has not been reported whether there are correlations between the key flowering regulators and auxin in wheat.

Given that, the *LBD* gene family served as essential transcription factors to regulate multiple plant growth and development processes, including heading date (Wang et al., 2013; Xu et al., 2016). In previous research, *MdLBD11* in apple is classified into class I subclade, and overexpression of *MdLBD11* in *Arabidopsis* results in delayed flowering (Wang et al., 2013). OsLBD37 and OsLBD38 in rice act as class II type LBD proteins, and overexpression of either *OsLBD37* or *OsLBD38* results in delayed heading date and increased yield. In this work, we found that *TaLBD16-4D* might be involved in auxin pathway and overexpression of *TaLBD16-4D* in wheat also led to early heading. Notably, the transcript level of *TaLBD16-4D* was induced by 2,4-D, and the endogenous IAA content in both roots and shoots of *TaLBD16-4D* overexpression plants was significantly increased compared to those of WT (**Figure 3A**). Moreover, the expressions of *TaGI*, *TaCO1*, *TaHd1*, *TaVRN1*, *TaVRN2*, and *TaFT1* could be induced by IAA treatment, respectively (**Supplementary Figure 6**). For the critical regulators in the vernalization pathway, the expression level of activator *TaVRN1* was greatly upregulated in *TaLBD16-4D* OE lines, and the expression of the inhibitor *TaVRN2* was remarkably downregulated (**Figure 5**), which collaborated to promote the expression of *TaFT1* and led to early flowering. Meanwhile, the pivotal genes in the photoperiod pathway studied here included *TaGI*, *TaCO1*, *TaHd1*, and *TaFT1* (Shimada et al., 2009; Shaw et al., 2013; Hu et al., 2020)*. TaLBD16-4D* might positively influence the expression of *TaGI*, *TaCO1*, and *TaHd1* under long-day conditions, which resulted in an upregulated expression of *TaFT1* to promote wheat flowering. Collectively, we deduced that *TaLBD16-4D* promotes wheat heading under long-day conditions probably through auxin-regulated flowering pathways. Therefore, it is necessary to further explore the molecular mechanism on how *TaLBD16-4D* participates in the auxin-mediated flowering pathway in wheat.

## Data availability statement

The original contributions presented in the study are included in the article/**Supplementary Material**. Further inquiries can be directed to the corresponding authors.

## Author contributions

YZ and JX designed the research. HW, XH, XS, XF, HC, SC, XW, YL, WG, and XL conducted the experiments. HW wrote the first draft. HW, XH, XF, and XS prepared the tables and figures. YZ and JX revised the manuscript. All authors read and approved the manuscript.

## Funding

This work was supported by the Natural Science Foundation of China (31901546), the Key Research and Development Project of Shandong Province (2021LZGC025), the Agricultural Variety Improvement Project of Shandong Province (2019LZGC016), and the Foundation for High-level Talents of Qingdao Agriculture University (6631119057).

## Conflict of interest

The authors declare that the research was conducted in the absence of any commercial or financial relationships that could be construed as a potential conflict of interest.

## Publisher’s note

All claims expressed in this article are solely those of the authors and do not necessarily represent those of their affiliated organizations, or those of the publisher, the editors and the reviewers. Any product that may be evaluated in this article, or claim that may be made by its manufacturer, is not guaranteed or endorsed by the publisher.
