# Supplementary material for: Overexpression of TaLBD16-4D alters plant architecture and heading date in transgenic wheat
Source: Front Plant Sci. 2022 Sep 21;13:911993. doi: 10.3389/fpls.2022.911993 (PMC9533090; doi:10.3389/fpls.2022.911993)
Supplement: Supplementary file 1 [file DataSheet_1.docx]

Supplementary Material

Overexpression of *TaLBD16-4D* Alters Plant Architecture and Heading Date in Transgenic Wheat


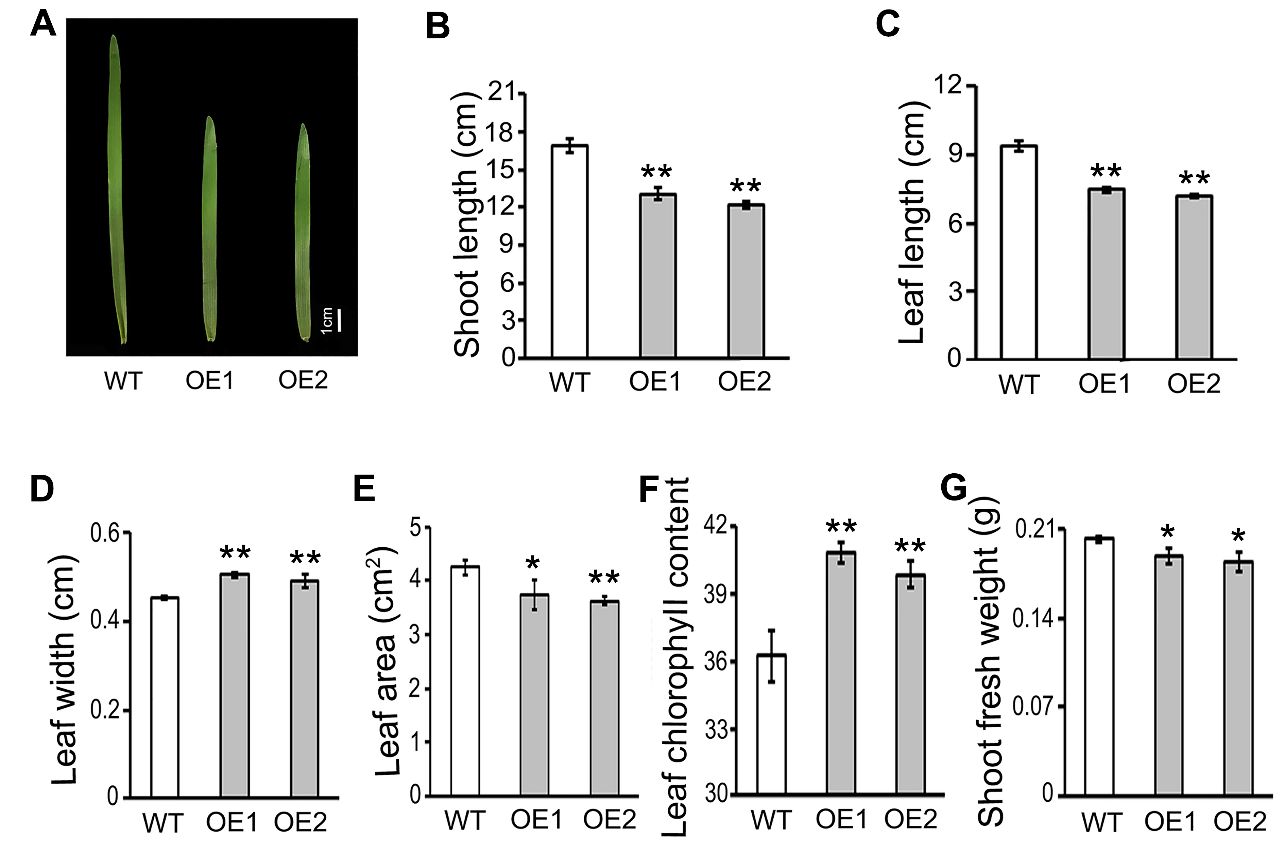


**Supplementary Figure 1** Morphological changes in two-leaf wheat seedlings of *TaLBD16-4D*-overexpressing plants cultured in pots in a greenhouse. **(A)** The first leaf morphology of WT and two OE lines. **(B)** to **(G)** ANOVA of shoot length **(B)**, leaf length **(C)**, leaf width **(D)**, leaf area **(E)**, leaf chlorophyll content **(F)**, and shoot fresh weight **(G)**. Values are all shown as mean ± SD (n = 3). The seedlings number of each replicate was at least 10 plants. Statistical differences between WT and two OE lines are indicated by asterisks and were determined using Student *t*-test: **P* < 0.05 and ***P* < 0.01.

**Supplementary Figure 2** *TaLBD16-4D*-overexpressing transgenic plants had less nodes and shortened internodes. Values are all shown as mean ± SD (n = 3). Five individual plants for each replicate were used for observation. Statistical differences between WT and two OE lines are indicated by asterisks and were determined using Student *t*-test: ***P* < 0.01.


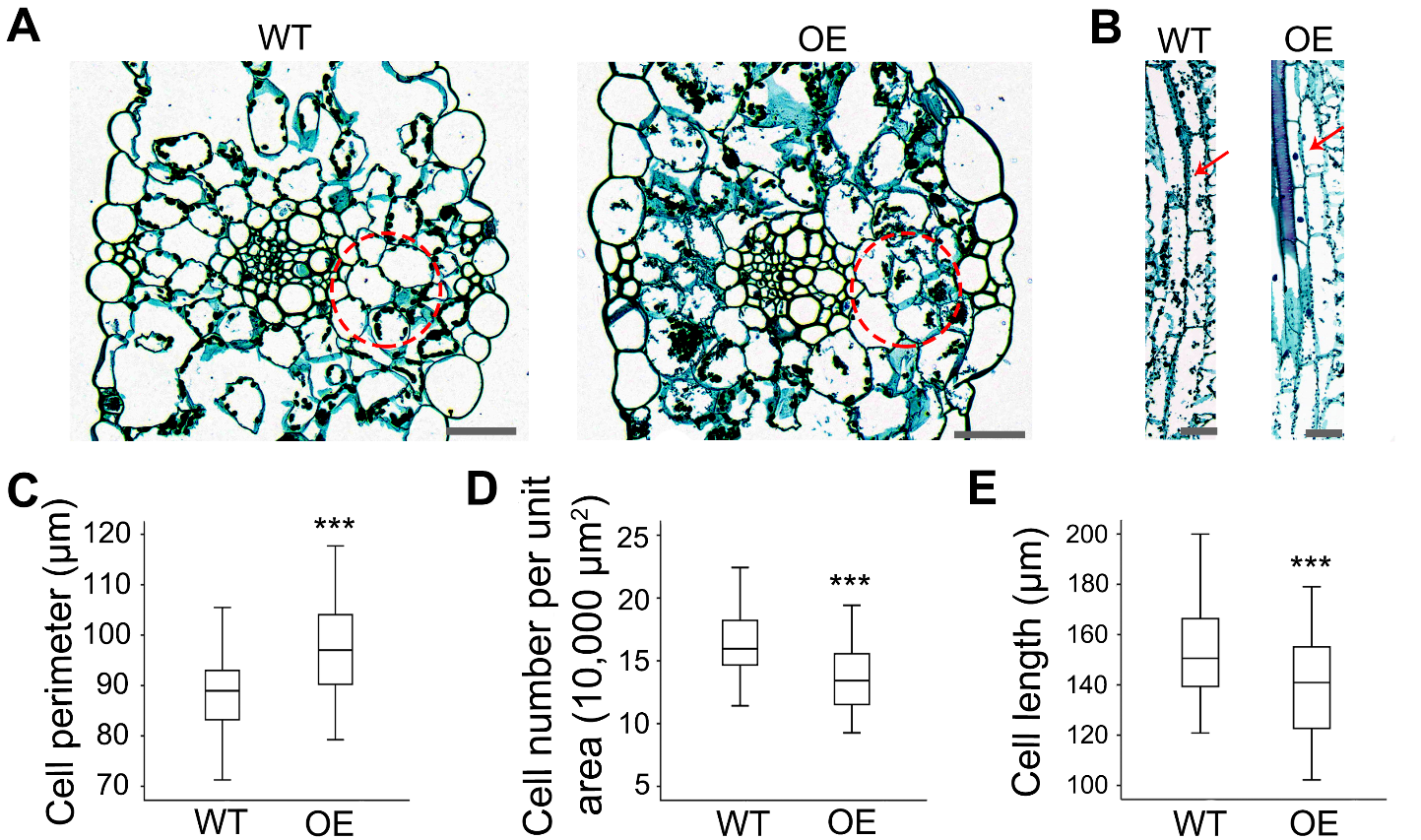


**Supplementary Figure 3** *TaLBD16-4D* regulation of cell size to control wheat leaf size. **(A)** Transverse section of the first leaf of WT and transgenic line (OE). Dotted circles in red indicate the major cell types selected for cell counting and measurement. **(B)** Longitudinal section of the first leaf of WT and OE line. Red arrow heads indicate the major cell types selected for cell measurement. Cell size **(C)** and Cell number per unit area **(D)** were examined in a. Cell length **(E)** was measured in b. For cell perimeter determination, representative cells within each of the red circle regions were selected for cell size measuring. For cell numbers (per unit area) counting, a total area of 10,000 um^2^ for each sample was investigated. For cell length determination, representative cells marked by red arrow heads were used for cell length measuring. Values are means ± SE (n＞50). Asterisks indicate statistically significant differences between the WT and *TaLBD16-4D*-overexpression plants determined using Student’s t-test. *** indicates significant differences at the 0.001 level. Bar = 50 μm


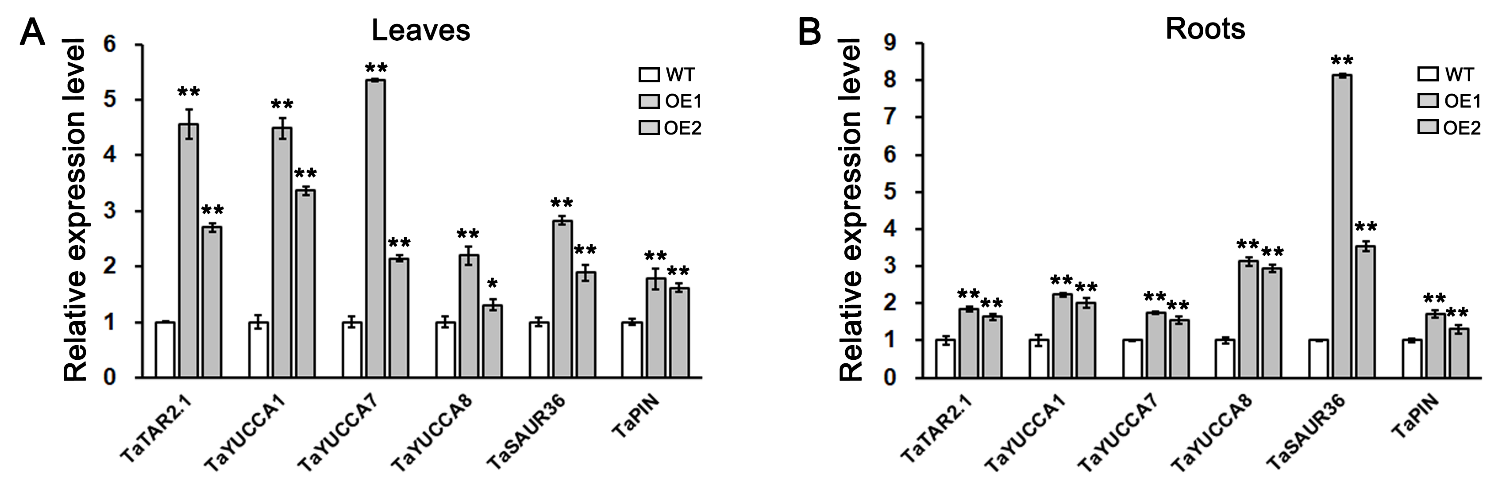


**Supplementary Figure 4** Expression patterns of genes involved in the auxin biosynthesis and transport in leaves (A) and roots (B) of WT and *TaLBD16-4D*-overexpressing transgenic plants. The expression of *TaActin* was used to normalize mRNA levels. The relative levels of each gene were normalized with the respective gene in WT set as 1. The values are means (±SE) of three biological replicates. Asterisks indicate statistically significant differences (**P* < 0.05, ***P* < 0.01 by two-sided *t* test).


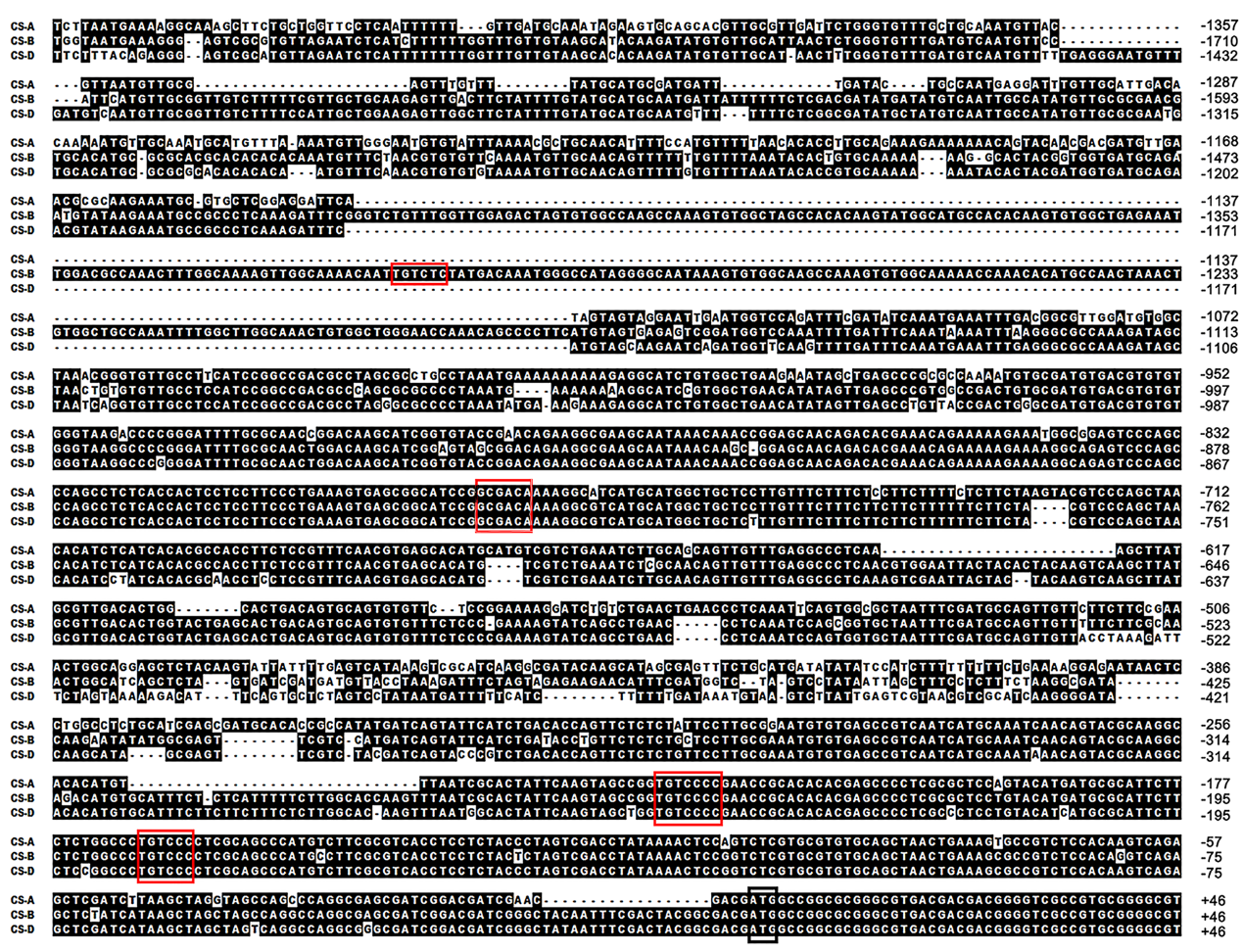


**Supplementary Figure 5** Sequence alignment of the promoter region of three *TaLBD16* homoeologues from Chinese spring (CS). The promoter of three *TaLBD16* homoeologues was designated CS-A, CS-B and CS-D, respectively. The promoter region is relative to the upstream of the ATG translation initiation codon. The position of the *TaLBD16* promoter is relative to the first base of the ATG codon. Black box indicates the start codon. Red boxes represent the putative auxin-responsive element (AuxRE).


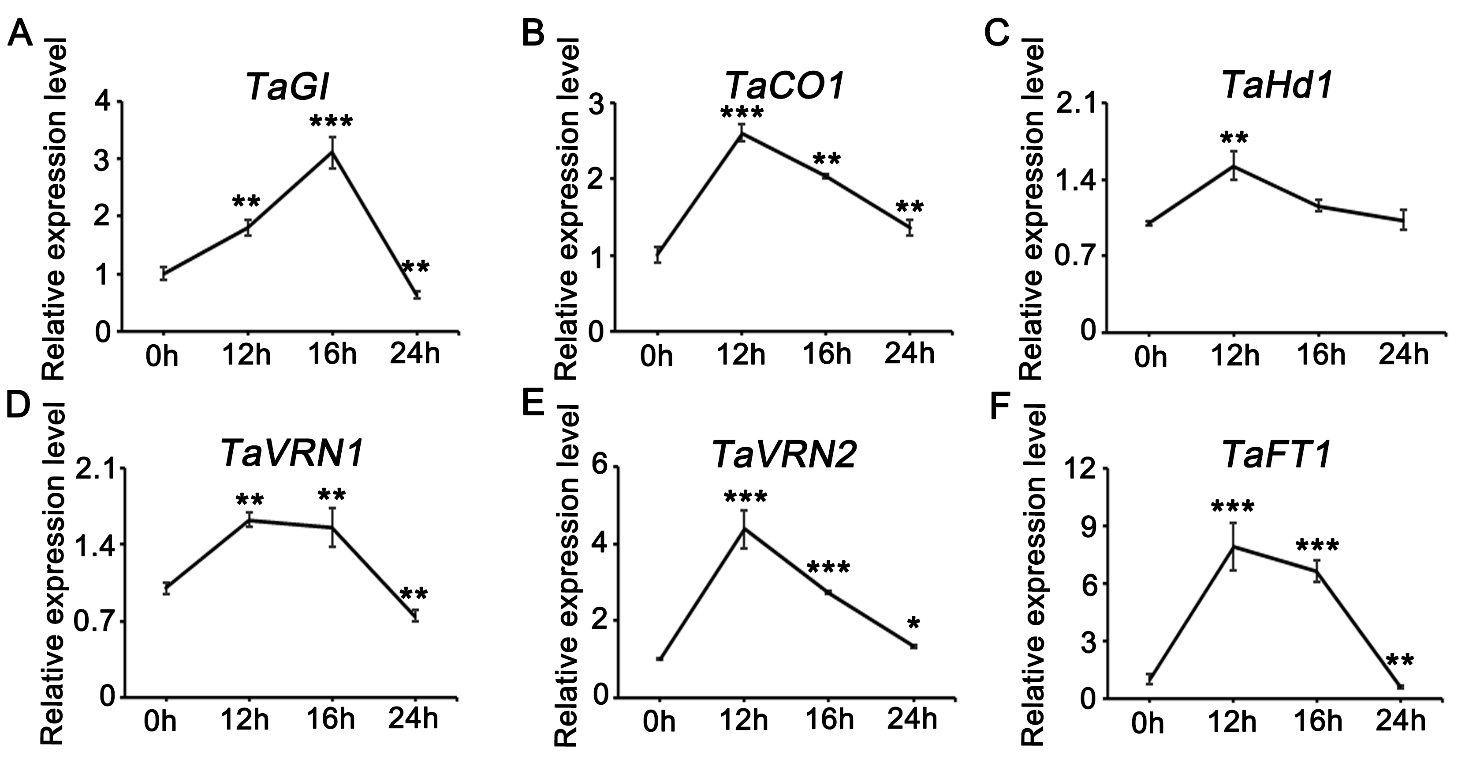


**Supplementary Figure 6** Expression of flowering-related genes in response to IAA. Plants with the third leaves were treated with 0.1 μM IAA for the indicated times. Expression levels of *TaGI* (A), *TaCO1* (B), *TaHd1* (C), *TaVRN1* (D), *TaVRN2* (E) and *TaFT1* (F) in WT normalized to that of *TaActin*. Expression in 0 h was set to one. Error bars indicate standard deviation derived from three biological replicates. Asterisks denote statistically significant differences (**P* < 0.05, ***P* < 0.01, ****P* < 0.001 by two-sided *t* test).

**Supplementary Table 1** Primers used in this study for gene expression analysis by qRT-PCR

| **Primers** | **Primer sequence (5' → 3')** |
| --- | --- |
| *TaGI*-L | GAAGGTCAGAAGATGTGGAGAGTCAAC |
| *TaGI*-R | GGCAGCGGATGGTAGGTGATAG |
| *TaCO1*-L | TTGATCCTTGGCCGTGCTT |
| *TaCO1*-R | GCACCACTTGTAGGGGCAGA |
| *TaHd1*-L | CCAGTACCTACACAGCTTCCA |
| *TaHd1*-R | GCCTGCTTCTTCTCCTTGT |
| *TaVRN1*-L | GGAGAGGTCACTGCAGGAGGA |
| *TaVRN1*-R | GCCGCTGGATGAATGCTG |
| *TaVRN2*-L | GGCCTGCCCATTATCCC |
| *TaVRN2-*R | GCGAAGCTGGAGATGATGG |
| *TaFT-*L | GCCAGAACTTCAACACCAGG |
| *TaFT-*R | TCAATTGTACATCCTCCTGCC |
| *TaIAA9-*F | CGAGCTTTCATGCACCTGTT |
| *TaIAA9-*R | CATTGCTTGCTCCTTGAGGT |
| *TaIAA21-*F | CTACCGACAGCCTCCTCAT |
| *TaIAA21-*R | TGCCGTCGTCGGACCTGT |
| *TaIAA23-*F | CTATGAGGACACCATTGACT |
| *TaIAA23-*R | TTGGGAGCAGGTGGCTTCT |
| *TaIAA24-*F | CCTCCAAGGTTCAGGTGGT |
| *TaIAA24-*R | CGTACAGCCTCCCTCTCTT |
| *TaIAA26-*F | CCATTGACATTGGTAGCTTCT |
| *TaIAA26-*R | CTCCTCCCATGGGTCATCA |
| *TaIAA19-*F | GTCGAGGAGAGCGACAAGAT |
| *TaIAA19-*R | CTGGAAGCAGCTCTTCCTGT |
| *TaIAA1-*F | CTTCTTCCATGGACAGCTGT |
| *TaIAA1-*R | GAGGTGAAGGTCCGTGCTT |
| *TaIAA28-*F | GAGGTGGTGGAGGAGAACT |
| *TaIAA28-*R | GAGGAGGACACGGAGGAGT |
| *TaIAA3-*F | TCGTCCAAGACCAAGTTCGT |
| *TaIAA3-*R | GTGAGGTGGGAGATGAACTT |
| *TaIAA14-*F | GGATGCAGCTGGGAAGATGT |
| *TaIAA14-*R | GCCTCTGTCTGCATCTTTGT |
| *TaIAA33-*F | CGTGCAAGCTCGTGAAGGT |
| *TaIAA33-*R | CGTCCCTGTCCTCGTAGGT |
| *TaARF7/19.1-*F | GCTTGTTGGAAAATGCAC |
| *TaARF7/19.1-*R | GAATGTACGCATCCTCTGTGG |
| *TaARF7/19.2-*F | GCAACTTCAGTTTGGCTTTCC |
| *TaARF7/19.2-*R | CGCACCAAATGACTGTGAAAC |
| *TaARF7/19.3-*F | TTCTGGCCAGAAGCAGCATAC |
| *TaARF7/19.3-*R | TCCCTGAAAGACGGTATGACTC |
| *TaARF7/19.4-*F | GTCCACCACCATTTTTCAGG |
| *TaARF7/19.4-*R | CCAGCTGGTAGCATCTGAGGA |
| *TaLBD16-4D-*L | TCCATGACGTCCACGCAGA |
| *TaLBD16-4D-*R | GCCGTAGCCGCAGTACATC |
| *TaPUCHI-*F | GACCCGACCACCAAGGAG |
| *TaPUCHI-*R | GAAGGGCGGGTAGTTGTTGT |
| *TaGATA2-*F | CCAACGAATGATCACCAAGG |
| *TaGATA2-*R | CAAGGACCACTCCTCCACAA |
| *TaTAR2.1-*L | TGTGTCTGTCGCATTGAATGTC |
| *TaTAR2.1-*R | ATGTTGCTGATCAGGAGAGATGAC |
| *TaYUCCA1-*L | CGTGCTACAGGAGGTCTATCTC |
| *TaYUCCA1-*R | CCAAGTCACATGAGGAACATCT |
| *TaYUCCA7-*L | TCCACGACAACACCACTTTATA |
| *TaYUCCA7-*R | CCTCTGCATTGCTTTAACAGTT |
| *TaYUCCA8-*L | TTATTACTGTAGCTAAGGGGCG |
| *TaYUCCA8-*R | TACAAGATGGTGAAATGAGCCT |
| *TaSAUR36-*F | TCTCTTCCTCGTCCGACTCC |
| *TaSAUR36-*R | CTTCGTCTTTTTCCCGCGTC |
| *TaPIN-*L | GGACCCGAACAACAATGGGA |
| *TaPIN-*R | CAGACGAGCGACCAGATGAG |
| *TaActin-F* | TATGCCAGCGGTCGAACAAC |
| *TaActin-R* | GGAACAGCACCTCAGGGCAC |
|  |  |
